# Identification of potential diagnostic markers of prostate cancer and prostatic intraepithelial neoplasia using cDNA microarray

**DOI:** 10.1054/bjoc.2001.1816

**Published:** 2001-06

**Authors:** J H Bull, G Ellison, A Patel, G Muir, M Walker, M Underwood, F Khan, L Paskins

**Affiliations:** Enabling Science and Technology, AstraZeneca, Cheshire SK10 4TG, UK; Department of Urology, King's College Hospital, Denmark Hill, London SE5 9RS, UK; Department of Urology; Department of Histopathology, Imperial College of Science, Technology and Medicine at St. Mary's Hospital, Praed Street, London W2 1NY; Department of Urology, Queen Elizabeth Building, Glasgow Royal Infirmary, Glasgow, G31 2ER

**Keywords:** array, carcinoma, PIN, BPH, benign prostatic hyperplasia, diagnosis

## Abstract

The identification of novel genes or groups of genes expressed in prostate cancer may allow earlier diagnosis or more accurate staging of the disease. We describe the assembly and use of a 1877-member microarray representing cDNA clones from a range of prostate cancer stages and grades, precursor lesions and normal tissue. Using labelled cDNA from tumour samples obtained from TURP or radical prostatectomy, analysis of expression patterns identified many up-regulated transcripts. Cell lines were found to over-express fewer genes than diseased tissue samples. 17 known genes were found to over-express more than 4-fold in 4 or more cancers out of 15 cancers. Only 2 genes were over-expressed in 6 out of 15 cancers or more, whilst no genes were consistently found to be over-expressed in all cancer samples. Novel prostate cancer associations for several well characterized genes or full length cDNAs were identified, including *PLRP1, JM27, human UbcM2, dynein light intermediate chain 2* and *human homologue of rat sec61*. Novel associations with high-grade PIN include: *breast carcinoma fatty acid synthase* and cDNA *DKFZp434B0335*. We shortlist and discuss the most significant over-expressed genes in prostate cancer and PIN, and highlight expression differences between malignant and benign samples. © 2001 Cancer Research Campaign http://www.bjcancer.com


*British Journal of Cancer* (2001) **84**(11), 1512–1519
© 2001 Cancer Research Campaign
doi: 10.1054/ bjoc.2001.1816, available online at http://www.idealibrary.com on IDEAL®

http://www.bjcancer.com

### Identification of potential diagnostic markers of prostate cancer and prostatic intraepithelial neoplasia using cDNA microarray

**JH Bull[1]*, G Ellison[1], A Patel[3], G Muir[2], M Walker[4], M Underwood[5], F Khan[1] and L Paskins[1†]**

[1]Enabling Science and Technology, AstraZeneca, Cheshire SK10 4TG, UK; [2]Department of Urology, King's College Hospital, Denmark Hill, London SE5 9RS, UK; [3]Department of Urology; [4]Department of Histopathology, Imperial College of Science, Technology and Medicine at St. Mary's Hospital, Praed Street, London W2 1NY; [5]Department of Urology, Queen Elizabeth Building, Glasgow Royal Infirmary, Glasgow, G31 2ER

**Summary** The identification of novel genes or groups of genes expressed in prostate cancer may allow earlier diagnosis or more accurate staging of the disease. We describe the assembly and use of a 1877-member microarray representing cDNA clones from a range of prostate cancer stages and grades, precursor lesions and normal tissue. Using labelled cDNA from tumour samples obtained from TURP or radical prostatectomy, analysis of expression patterns identified many up-regulated transcripts. Cell lines were found to over-express fewer genes than diseased tissue samples. 17 known genes were found to over-express more than 4-fold in 4 or more cancers out of 15 cancers. Only 2 genes were over-expressed in 6 out of 15 cancers or more, whilst no genes were consistently found to be over-expressed in all cancer samples. Novel prostate cancer associations for several well characterized genes or full length cDNAs were identified, including *PLRP1, JM27, human UbcM2, dynein light intermediate chain 2* and *human homologue of rat sec61*. Novel associations with high-grade PIN include: *breast carcinoma fatty acid synthase* and cDNA *DKFZp434B0335*. We shortlist and discuss the most significant over-expressed genes in prostate cancer and PIN, and highlight expression differences between malignant and benign samples. © 2001 Cancer Research Campaign http://www.bjcancer.com

**Keywords**: array; carcinoma; PIN; BPH; benign prostatic hyperplasia; diagnosis

Prostate cancer is now the most commonly diagnosed cancer in males in the US, and is the second leading cause of cancer death (Landis et al, 1998). Recent increases in detection rates, through serum PSA testing and increased public awareness, have resulted in larger numbers of patients being diagnosed, particularly in early stages of the disease (Catalona et al, 1993).

Markers are needed to help stratify the disease spectrum. Raised PSA levels are not specific for malignant disease, and efficient second line diagnostic methods are needed to deal with the increased numbers of individuals being identified (Crawford et al, 1996). There is also intense interest in the efficient detection of precursor lesions such as high-grade PIN, which significantly increases the likelihood of cancer development (Haggman et al, 1997). Following initial diagnosis, inadequate tools and poor predictive methods handicap prostate cancer management. Currently, microscopic analysis of tissue by a pathologist is the established method (Gleason, 1993). However, histopathology may be expensive and time consuming, and is not of sufficient sensitivity to be predictive for the majority of patients who fall into the intermediate grades (Menon, 1997). It is hoped that advances in genetics and gene expression will allow more accurate assessment of tumour aggressiveness, perhaps by use of a panel of tumour biology markers reflecting proliferation, angiogenesis, loss of cellular adhesion, invasion, likelihood of metastasis and avoidance of cell death (Hegarty et al, 1999).

These problems may be addressed using a genomics-based approach investigating global gene expression changes in clinical samples using microarrays (Sagar, 1997; Burczak et al, 1998). With suitable databases and bioinformatics tools, candidate genes can be selected following in silico analysis for favourable tissue distribution, secretion signals and other features, allowing empirical design of microarrays for candidate marker screening. Gene expression profiling is being increasingly used to analyse hundreds or thousands of genes simultaneously in cancer cell lines (Bertucci et al, 1999), and diseased and normal tissue (Zhang et al, 1997; Alon et al, 1999; Wang et al, 1999). Clustering analysis of gene expression data can provide novel insights into disease, for example molecular definition of subtypes of leukaemia, providing a tool for an important diagnostic problem (Golub et al, 1999).

To identify potential candidate prostate cancer markers we assembled a custom microarray for analysis of prostate tissue, biasing the clone choice on our array towards genes that are expected to express at higher levels in cancer. The frequency of clones in tissue-specific cDNA libraries in the proprietary Incyte databases, which typically rank genes according to frequency in a given library, was taken to reflect their true abundance in tissues. Quantitative electronic subtraction of cDNA libraries (e.g. cancer minus normal, late-stage minus early-stage) was used to increase the odds of including relevant genes on the array, which could then be checked by hybridization to diseased prostate cDNA. In

*Received 4 August 2000*
*Revised 22 February 2001*
*Accepted 28 February 2001*

*Correspondence to:* JH Bull

*Current address: CMC International, King Edward Street, Macclesfield, Cheshire SK10 1AQ.
†Current address: A1-Biotech UK, Anachem Ltd., Charles St., Luton, Bedfordshire, LU2 0EB.

addition, in silico transcript imaging analysis was used to check specificity against other tissue types. Known cancer and prostate-associated genes were also included in the array.

Although down-regulated genes are of great biological interest, it is not convenient to assay their transcripts because biopsy or other samples may contain normal tissue to varying degrees, and disease tissue may form only a small part of the sample under investigation. Therefore we set out to identify strongly over-expressed genes, which may be detectable even in samples containing a minority of affected tissue. This analysis of normal, BPH, PIN and prostate cancer tissue provides a starting point for further investigation of candidate marker genes for diagnosing, staging and treating prostate cancer.

### MATERIALS AND METHODS

### Bioinformatics, databases and choice of cDNA clones

Genes (or, in the case of unknown genes, clone clusters) of potential interest for microarray were selected either because of a known association with prostate or cancer, or because of a relative abundance in tumour libraries compared to normal prostate. For the latter, manipulations were carried out using a proprietary database (LifeSeq) provided by Incyte Pharmaceuticals (Palo Alto, CA) which primarily contains grossly dissected and microdissected prostate cDNA libraries. Several public domain libraries including those from the Cancer Genome Anatomy Project are also available in this database, including PIN libraries. Using Incyte's electronic tools for transcript imaging (virtual Northern blot analysis) and library subtraction based on the BLAST algorithm, the array was biased towards over-expressed genes which might form useful diagnostic markers. This empirical approach was complemented by inclusion of known prostate- and/or cancer-associated genes from the literature. Physical clones for arraying were chosen from a proprietary collection supplied by Incyte Pharmaceuticals, many of which were sequence verified by Incyte. Where available, 2 different clones per gene were used, typically from the 3′ region of the transcript. An additional set of 11 'housekeeping' genes was also included. A master list of clones was maintained using Microsoft Excel software.

The transcript imaging tool was used to interrogate the Incyte Lifeseq Gold database for tissue distribution across 1113 cDNA libraries. All sequence annotation, gene ID and transcript imaging data presented in this paper was correct at the time of writing, according to Incyte Lifeseq Gold version 5.1, February 2000 release.

### PCR and clone arraying

Clones were assembled into 96-well microtitre plates containing selective growth medium, grown overnight at 37˚C. The cDNA inserts were amplified using universal primers for pINCY, pSPORT and pBluescript (TTGGGTAACGCCAGGGTTTTC-CCAGTCAC and CCCCAGGCTTTACACTTTATGCTTCCG-GC) at 35 pmol 50 $\mu l^{-1}$ reaction, in 96-well plates containing PCR buffer (1.05 units *Taq* DNA polymerase, 20 mM Tris-HCl (pH 8.3), 50 mM KCl, 1.5 mM $MgCl_2$ and 0.2 mM each dATP, dCTP, dGTP and dTTP) (AB gene, Epsom, UK). PCR conditions were 94˚C, 2 min, followed by 35 cycles of 94˚C, 1 min, 60˚C, 1 min and 72˚C 3 min followed by a final extension step of 72˚C for 10 min. PCR products were spotted onto Nytran + membrane (Schleicher and Schuell, Keene, NH) using an automated robotic system (Q-Bot, Genetix, Christchurch, UK) together with appropriate software. Null spots containing dye were used as a visual aid to assess array quality and orientation. The DNA was denatured using standard solutions and crosslinked to the membrane by ultraviolet irradiation (Stratagene Stratalinker, La Jolla, CA).

### Prostate RNA, labelling and hybridization conditions

Tumour samples were either from sections of radical prostatectomy specimens, or tissue from transurethral resection, with concurrent histopathological data. All clinical samples were obtained with the approval of the ethics committee of the hospital concerned. Tissue was flash frozen in liquid nitrogen and stored at –70˚C until needed. Culture of prostate carcinoma cell lines was performed under standard conditions. PC-3, DU 145 and LNCaP cells were cultured in RPMI medium (Gibco, Paisley, UK), 5% fetal calf serum (Gibco), 1% glutamate (Gibco). LNCaP cells were initially cultured in this medium for 48 h, then medium was replaced with charcoal-stripped medium, and cells incubated for a further 48 h, followed by 48 h in fresh medium with or without α-5-dihydrotestosterone. About 100 mg tissue or cells was used for RNA extraction using Trizol (Gibco). RNA integrity was checked by agarose gel electrophoresis. Normal prostate RNA was a mixture from 20–30 accident victims with no diagnosable prostate abnormality (Clontech, Palo Alto, CA). 15–20 μg total RNA was treated with 10 units DNase I (RNase free) (Roche Molecular Biochemicals, Lewes, UK) in a suitable volume, at room temperature for 15 min. EDTA was added to 2.5 mM, and the sample heated to 65˚C for 10 min to stop the reaction. If necessary, at this stage Microcon 30 columns (Amicon, Millipore, Bedford, UK) were used to concentrate the sample. 15–20 μg RNA was allowed to anneal to 0.5 μg of oligo (dT) primer 12–18 (Gibco) at 65–70˚C for 10 minutes. First strand labelling was performed with 1st Strand Labelling Buffer (Gibco) with the addition of dTTP, dATP and dGTP at a final concentration of 0.5 mM and dCTP at 50 μM, 30 mCi $\alpha$-$^{33}$P dCTP (Amersham Pharmacia Biotech) and 40 units RNase inhibitor (Roche). After 5 minutes at 42˚C, 200 units Superscript II enzyme (Gibco) was added and the mix was incubated at 42˚C for 1.5–2 hours. Unincorporated nucleotides were removed by centrifugation through a GFX column (Amersham Pharmacia Biotech) according to manufacturer's guidelines. The newly synthesized probe was denatured by boiling for 5 minutes and stored on ice. Microarray filters were wetted in Church hybridization solution (Church and Gilbert, 1984), and incubated with a further ~5 ml of this solution in a cylindrical bottle rotated in a hybridization oven at 65˚C for 4–5 h prior to the addition of fresh solution and denatured probe, which was mixed by quickly swirling. Hybridization was at 65˚C for ~ 16 h. Filters were washed in Church wash solution (Church and Gilbert, 1984) at 65˚C for $2 \times 20$ min.

### Image quantification and data analysis

Filters were covered in clingfilm wrap and exposed to low-energy phosphorimaging screens (Eastman Kodak, Rochester, NY) for 5–6 days, prior to phosphorimager scanning (Storm 830, Molecular Dynamics, Sunnyvale CA). Images were manipulated using ImageQuant software (Molecular Dynamics), and quantitative data for spot intensity extracted using Incyte LifeArray software, then exported to Microsoft Excel. The data were processed

© 2001 Cancer Research Campaign in an Excel workbook sheet designed to (a) calculate a local background value for each of 96 small 7 × 7 grid areas on the array, and discard data points within this area less than 2-fold this value, then using this output (b) normalize by calculating a ratio for each value against the mean value for housekeeping genes across the whole array. Data could then be directly compared between filters. A dataset for mean normal prostate was generated from 2 duplicate array hybridization experiments of 2 independent mixed batches of normal prostate RNA. The 2 normal prostate RNA batches comprised samples from 23 men (aged 23–64), and 47 men (aged 15–50), respectively, who were not diagnosed for prostate cancer (Clontech). Typically, data from tumour samples was exported to a second Excel workbook, and ratios generated against normal values. Genes could then be ranked for level of over-expression, and by assembling lists of ranked over-expressed genes, tumours compared and data amalgamated. In some cases, data for genes in the normal RNA were below the 2-fold background cut-off, and a ratio could not be generated. These genes were classified on/off (expressed in tumour, undetectable in normal).

### RESULTS

Samples of prostatic material were collected from well characterized patients with either BPH (mean age (range) 75 (66–84) years), PIN or prostate cancer (mean age (range) 68 (52–88) years) (sample details are given in Table 1). Array data were normalized to a representative selection of housekeeping genes, to allow comparison of expression levels between hybridization experiments. To generate a normal prostate dataset, mixed samples of normal prostate from individuals (aged 15–64 years) with no history of prostatic disease were used (Clontech), as normal prostate material was not available. The mean of 2 hybridization results for each of 2 different batches of normal prostate samples was taken. No differences of greater than 2-fold were observed between these pairs of normal prostate control filters. To evaluate overexpression compared to normal prostate tissue, results from cell culture, prostate cancer, PIN or BPH samples were ratioed against the control normal prostate dataset, and genes with values of greater than 1.5 × normal signal were considered to be over-expressed.

To evaluate overexpression of cancer relative to benign tumour, a mean of 11 BPH samples (Table 1) was taken as the control value, and results from cancer samples were ratioed against this. Subsequently, all of the genes identified as overexpressed were checked against the mean value for the set of 11 BPH samples. No BPH sample deviated by 1.5 fold or more from the mean for any of the genes of interest.

### Over-expressed genes are more common in prostate tissue than cells

The majority of genes found to be over-expressed in cancer, PIN and BPH samples were expressed in normal tissue also. A variable number of genes were expressed in diseased tissue but not detected

**Table 1** Details of prostate samples used in this study

| Sample | Origin/diagnosis | Sample designation | Gleason grade / other details | Clinical stage |
|---|---|---|---|---|
| A19 | TURP/PIN | PIN | PIN2 on biopsy | T0 |
| A39 | TURP/PIN | PIN | 6 (in 3% chips) + PIN2 | T0 |
| KC4 | CaP | CaP | unknown | unknown |
| A49 | radical/CaP | CaP – early | 4 + PIN2 | T2 N0 |
| G53 | TURP/CaP (20% of chips) | CaP – early | 2 + 2 | T1b |
| A53 | radical/CaP | CaP – early | 5 + PIN2 | T2 N0 |
| KC5 | TURP/CaP in ?% chips | CaP – early | 6 | T3 |
| A65 | radical/CaP | CaP – early | 6 + PIN2 | N0 |
| A48 | radical/CaP | CaP – early | 6 | N0 |
| A5 | radical/CaP | CaP – early | 3 + 3 + PIN2 | T2b N0 |
| A3 | radical/CaP | CaP – mid | Left= 3+4: Right = PIN | T3c N0 |
| KC1 | TURP/CaP in only 10% chips | CaP – mid | 8 | unknown |
| A46 | TURP/CaP | CaP – late | 9 | NX |
| A63L | radical/CaP | CaP – late | 9 + PIN2 | N1 |
| A63R | radical/CaP | CaP – late | 9 + PIN2 | N1 |
| A2 | TURP/CaP | CaP – late | 9 | unknown |
| KC3 | TURP/CaP in 90% chips | CaP – late | 9 received post-hormonal therapy | unknown |
| KC2 | TURP/CaP in nearly all chips | CaP – late | 5 + 4 | T4 |
| KB1 | TURP/BPH | BPH | N/A | T0 |
| KB11 | TURP/BPH | BPH | N/A | T0 |
| KB2 | TURP/BPH | BPH | N/A | T0 |
| KB3 | TURP/BPH | BPH | N/A | T0 |
| KB4 | TURP/BPH | BPH | N/A | T0 |
| KB5 | TURP/BPH | BPH | N/A | T0 |
| KB6 | TURP/BPH | BPH | N/A | T0 |
| KB7 | TURP/BPH | BPH | N/A | T0 |
| KB8 | TURP/BPH | BPH | N/A | T0 |
| KB9 | TURP/BPH | BPH | N/A | T0 |
| KB10 | TURP/BPH/prostatitis | BPH/prostatitis | N/A | T0 |

Range of samples used in this study showing histological data and tumour stage where known/appropriate. The Gleason grading system is widely used as it relates differentiation of cancer, and correlates with prognosis. The scale used is 2–10 (Gleason, 1992). Tumour stage details are according to the TNM classification system: T0 indicates no evidence of primary tumour; T1–T2 tumours are confined to the prostate, T3 tumours extend through the prostatic capsule, T4 indicates invasion of adjacent structures, N0 indicates no regional lymph node metastasis; N1 indicates limited lymph node involvement (full details of the TNM system are in Jones and Smith, 1994).

*© 2001 Cancer Research Campaign*

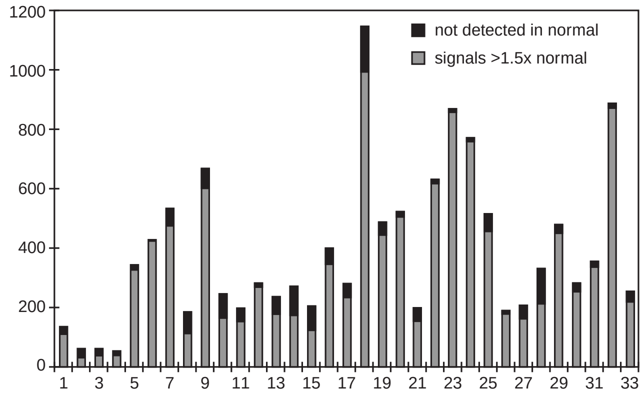

**Figure 1** Genes over-expressed in various sample types compared to normal prostate. The graph plots the total number of over-expressed genes for each prostate sample (1–33); cell lines (1–4); LNCaP, LNCaP treated by androgen deprivation, PC-3, DU 145; PIN (5,6), TURP chips containing BPH (7–17), TURP chips containing BPH and cancer (18–20), TURP chips containing cancer, and radical prostatectomy segments (20–33)

in normal tissue, but these genes were always in a minority compared to genes detected in normal but over-expressed in tumour (Figure 1). In contrast, prostate cancer cell lines displayed a high proportion of genes whose expression was not detected in normal prostate. Compared to normal prostate, LNCaP cells over-expressed roughly twice as many genes as DU-145, PC3 and LNCaP cells deprived of androgen (Figure 1 and Table 2). However, samples of prostate containing or comprising PIN, BPH or cancer consistently displayed much higher levels of global over-expression than the cell systems, typically in the range 200–600 genes (Figure 1). There is no strong difference in numbers of over-expressed genes between 2 PIN, 11 BPH and 16 cancer tissue samples (Table 2).

**Table 2** Levels of overexpression in sample types

| | Cell lines | PIN | TURP/BPH | TURP/some cancer | Cancer |
|---|---|---|---|---|---|
| Samples tested | 4 | 2 | 11 | 3 | 13 |
| Mean (SD) over-expressed genes | 75.2 (37.2) | 381.5 (57.3) | 315.5 (155.5) | 715.7 (371.4) | 456.3 (255.8) |

Mean numbers of genes over-expressed in different types of prostate material, relative to normal prostate tissue.

### Over-expressed genes in cancer

Table 3 lists 17 genes most commonly over-expressed in prostate cancer samples compared to normal prostate samples. In addition

**Table 3** Over-expressed genes in prostate cancer

| Gene identity/hit | gi number | Hits | Mean ratio/normal | Putative function/ cancer association | References |
|---|---|---|---|---|---|
| *CC chemokine gene cluster* | g3719360 | 5 | 6.9 | putative unknown gene in cluster | Nomiyama et al, 1999 |
| *cystatin SA-I* | g337751 | 5 | 6.8 | thiol protease inhibitor | Al-Hashim et al, 1988 |
| *autoantigen calreticulin* | g179881 | 4 | 7.5 | calcium binding protein | Rokeach et al, 1991 |
| *pancreatic lipase related protein PLRP1* | g187229 | 5 | 6.6 | unknown association | Giller et al, 1992 |
| *coagulation factor V* | g182411 | 6 | 6.3 | blood clotting | Jenny et al, 1987 |
| *FYN binding protein* | g2232150 | 4 | 5.4 | T-cell function | da Silva et al, 1997 |
| *JM27* | g3114821 | 4 | 6.8 | soares placenta | unpublished |
| *mouse ubiquitin-conjugating enzyme UbcM2* | g5114058 | 4 | 5.9 | CD34+ cell | unpublished |
| *PA26-T1 nuclear protein* | g1329 | 4 | 5.5 | non p53-induced gene transcript in human Saos-2 osteosarcoma cells | Buckbinder et al, 1994 |
| *cytochrome P1-450* | g181275 | 5 | 5.9 | protection against chemical carcinogenesis | Jaiswal et al, 1985, Talalay, 1989 |
| *nucleolin* | g189305 | 5 | 5.5 | nucleolar phosphoprotein involved in the synthesis and maturation of ribosomes | Srivastava et al, 1990 |
| *p78 cell cycle regulated factor* | g2384716 | 4 | 7.1 | proliferation-related nucleolar protein found at much higher levels in most human malignant tumors | Ren et al, 1998 |
| *p97* | g1857236 | 6 | 20.7 | translational regulator, thought to be general repressor | Imataka et al, 1997 |
| *dynein light intermediate chain 2* | g2665835 | 4 | 5.3 | Cytoplasmic dynein is a multi-subunit complex involved in retrograde organelle transport and some aspects of mitosis | unpublished |
| *Rat sec61 homolog mRNA* | g5106794 | 4 | 4.3 | protein transport in NB4 cell line | unpublished |
| *cDNA DKFZp564C0362* | g5262486 | 4 | 6.6 | novel mRNA from fetal brain | unpublished |
| *SRF accesory protein 1A (SAP-1) s22 1992* | g429185 | 4 | 6.5 | binds to serum response factor DNA sequence. Also homology to cadherin-10 | Dalton and Treisman, 1992 |

Genes over-expressed in 4 or more cancer samples from a total of 15. Figures are mean fold signal relative to mean normal prostate (where normal prostate value was below the 2-fold background threshold, no normal value was available, so the figure given is fold signal over 2-fold background – see section 2.4). The mean value is calculated for a single clone spot per filter (that which gave the highest number of positives across the samples).

© 2001 Cancer Research Campaign to several blood cell-related protein genes which may be associated with host response to tumour presence (e.g. *FYN binding protein* and *human UbcM2* genes) the list contains several genes and full length cDNAs that are likely to be expressed in the prostate tissue and may constitute specific markers of cancer. These include the strongly over-expressed *p97* and *p78* genes. A novel association with prostate cancer is apparent for the full-length cDNA *DFKZp564CO362*, which encodes an uncharacterized protein. In addition, this is the first report of a prostate cancer association for the genes *PLRP1, JM27, human UbcM2, dynein light intermediate chain 2* and *human homologue of rat sec61*.

Following collation of these data, the transcript imaging tool was used in the Incyte Lifeseq Gold database to check tissue distribution across 1113 cDNA libraries. Examples of genes for which this data reflected and supported array data are *CC chemokine* and *dynein light intermediate chain 2*. *CC chemokine* tended to be represented in prostate cancer and PIN libraries, but not normal prostate or other tissue types. *Dynein light intermediate chain 2* was present in many tissue types, but within the set of prostate libraries tended to be over-represented in cancer. For other genes, transcript imaging analysis gave mixed results which did not always reflect array data: *JM27* was present almost exclusively in prostate libraries, but in both normal and disease types; in contrast *FYN binding protein* and *cytochrome p-1450* were not present in any prostate libraries. Their presence on the array was presumably fortuitously due to reallocation to new gene clusters by Incyte following our initial clone selection at the array construction stage (see Discussion). The rest of the markers characterized by the microarray results appeared in many tissue libraries and had no obvious prostate cancer association as indicated by transcript imaging data.

### Over-expressed genes in high grade PIN

Table 4 lists 9 genes most commonly over-expressed in high-grade PIN relative to normal prostate. Again, host response genes feature (e.g. *SRp40–1* and *CD–24*), but are outnumbered by genes of likely prostatic origin, e.g. *k-ras*. Novel associations with prostate cancer include *breast carcinoma fatty acid synthase* and the full length cDNA *DKFZp434B0335*. Transcript imaging analysis showed wide tissue library distribution for all these genes, but an association with prostate cancer was particularly strong for *fatty acid synthase* and *TAT interactive protein* genes.

### Over-expression in cancer compared to benign tumour

6 transcripts were over-expressed in at least 5 out of 13 prostate cancer samples compared to mean BPH transcript level (Table 5).

**Table 4** Genes over-expressed in PIN

| Gene identity | gi number | PIN 19 | PIN 39 | Function/association | References |
|---|---|---|---|---|---|
| *DKFZp434B0335* | g5911996 | 7.12 | 6.83 | adult testis | unpublished |
| *IGFBP 4* | g33265191 | 5.99 | 5.61 | bone cells | Zazzi et al, 1998 |
| *splicing factor SRp40-1* | g1049079 | 6.25 | 6.52 | T-cell activation | Screaton et al, 1995 |
| *DiGeorge syndrome critical region, centromeric end* | g137775 | 4.94 | 3.21 | unknown | Gong et al, 1996 |
| *Human lupus p70 (Ku) autoantigen* | g178649 | 4.95 | 7.54 | unknown | Reeves and Sthoeger, 1989 |
| *fatty acid synthase* | g91531 | 5.72 | 3.85 | breast carcinoma | unpublished |
| *TAT interactive protein* | g1657981 | 4.6 | 6.26 | interacts with HIV protein | Kamine et al, 1996 |
| *k-ras* | g1815608 | 4.02 | 2.05 | proto-oncogene | Kahn et al, 1987 |
| *CD-24 signal transducer* | g500848 | 5.02 | 6.47 | B-cell activation | Kay, 1991 |

Genes over-expressed more than 2-fold, in common between 2 PIN samples (figures given are ratios against normal prostate; representative data are given for one of 2 identical spots on the array).

**Table 5** Genes over-expressed in prostate cancer relative to BPH

| Gene identity | gi number | Hits | CaP/normal ratio | Function | References |
|---|---|---|---|---|---|
| *G(i) protein alpha-subunit* | g31743 | 6 | 2.2 | Increased levels associated with monocyte differentiation. | Didsbury et al, 1987 |
| *CC chemokine* | g3719360 | 7 | 3.8 | | Nomiyama et al, 1999 |
| *metallopeptidase PRSM1* | g1354930 | 8 | 2.8 | Growth factor activation and extracellular matrix synthesis and degradation. Found in cultured osteosarcoma cells | Scott et al, 1996 |
| *DKFZp586D091* | g4884127 | 5 | not detected in normal or BPH | Adult uterus | unpublished |
| *laminin B2 chain* | g186962 | 5 | not detected in normal or BPH | Epithelial cell basement membrane | Kallunki et al, 1991 |
| *repressor of estrogen receptor activity (REA)* | g5020252 | 5 | not detected in normal or BPH | May play an important role in determining the sensitivity of oestrogen target cells in BC | Montano et al, 1999 |

Markers increased by more than 1.5-fold in 5 or more cancer samples out of 15, relative to mean value for BPH. CaP/normal values are mean expression levels for those samples in which signal >1.5 × normal, or >1.5 × mean of 13 BPH samples. 3 genes were found to be expressed in cancer but not detected in BPH.

 © 2001 Cancer Research Campaign

None of these 6 genes was expressed by more than 1.5 × mean BPH level in any of the 11 BPH samples listed in Table 1. Only one of these genes, *CC Chemokine*, features in the set of genes identified as over-expressed in 4 or more cancers compared to normal prostate (Table 3). Levels of over-expression in cancer relative to BPH are much lower than cancer relative to normal prostate (compare Tables 3 and 5), suggesting that most genes over-expressed in cancer are also over-expressed in BPH, and so may be of limited use in a molecular diagnostic situation necessitating differentiation between the 2 disease states. However, we detected 3 genes in 5/13 cancers which were not detected in any of 11 BPH samples used in this study. These are *DKFZp586D091* (a full length cDNA encoding a protein of unknown function), *laminin B2 chain*, and a *repressor of oestrogen activity (REA)*. Transcript imaging supported this data: *DKFZp586D091* was represented in only a few libraries per tissue type with the exception of nervous system, and at high frequency in only one prostate (tumour) library; *REA* was more ubiquitously and frequently represented, but was common in prostate tumour libraries and rare in normal and BPH. These 3 genes were not co-expressed in the same cancers (data not shown), but a combination of them could form the basis of a diagnostic test capable of detecting cancer in a prostate sample containing or comprising mostly BPH cells.

**Table 6** Comparative levels of gene expression in prostate cancer

| Gene identity | gi number | Mean expression level |
|---|---|---|
| *TAT interactive protein* | g1657981 | 15.9 |
| *myosin light chain kinase* | g1262344 | 4.12 |
| *ribosomal protein L35* | g34200 | 3.4 |
| ***histone H 3.3*** | g761715 | 3.37 |
| ***p97*** | g1857236 | 2.49 |
| *ribosomal protein L18* | g337492 | 2.21 |
| *fibroblast collagenase inhibitor* | g182482 | 2.03 |
| *p97* | g1857236 | 1.99 |
| *PSA* | g35720 | 1.95 |
| *metallopeptidase PRSM1* | g1354930 | 1.86 |
| *laminin B2* | g186962 | 1.53 |
| *steroid 5 alpha reductase* | g338468 | 1.47 |
| ***Novel human gene mapping to chromosome 1*** | g5834563 | 1.35 |
| *p27* | g2982672 | 1.34 |
| ***proteasome subunit HC5*** | g220025 | 1.32 |
| ***coagulation factor V*** | g182411 | 1.32 |
| *transforming growth factor alpha* | g37089 | 1.32 |
| *prostatic acid phosphatase* | g439664 | 1.31 |
| ***bcl-2*** | g179370 | 1.18 |
| *ribosomal protein L35* | g407422 | 1.16 |
| *metalloproteinase inhibitor* | g608128 | 1.14 |
| *nucleolin gene* | g189305 | 0.91 |
| ***cystatin SA-I*** | g337751 | 0.9 |
| ***CC chemokine*** | g3719360 | 0.81 |
| ***ublquitin activating enzyme E1*** | g340071 | 0.77 |
| ***JM27*** | g3114821 | 0.71 |
| ***putative protein kinase regulator*** | g3641524 | 0.58 |
| ***pre-mRNA splicing factor (PRP17)*** | g3123907 | 0.41 |

Relative levels of gene expression in cancer samples, showing details for a selection of gene transcripts detected in 4 or more /15 prostate tumours. Those not detected in normal prostate (i.e. signals < 2× background on control filters) are listed in **bold**. Mean expression levels are in arbitrary units.

### Relative levels of gene expression from array data

The data presented in Tables 3, 4 and 5 are in the form of ratios of transcript levels in malignant or pre-malignant disease compared to normal or benign disease. In order to compare relative levels of expression for genes of interest (including genes which were not detected in normal tissue, and for which an accurate over-expression ratio could not be generated) it was necessary to refer to normalized filter data on a sample to sample basis. Table 6 allows comparison of expression levels for many of the genes featured in Tables 3–5. For example, it can be seen that levels of *JM27* and *CC chemokine* (possible discriminatory marker for cancer versus normal cells – Table 3) transcripts are approximately 50% that of *laminin B2* (a possible discriminatory marker for cancer versus BPH cells – Table 5) and 35% that of prostate-specific antigen (PSA). These results will form the benchmark for further investigations with RT-PCR.

### DISCUSSION

We are interested in using molecular criteria to distinguish between different disease states within the prostate. Accordingly, we have focused on genes which exhibited higher than normal levels of expression in cancer and PIN, as indicated by tissue distribution data in silico. Following array analysis, our aim was to shortlist genes that could enable molecular diagnosis of cancer and/or PIN despite the quantities of normal and BPH tissue which may be present in a biopsy sample. The results we present form the starting point for further studies using accurate RNA quantitation methods such as RT-PCR (e.g. Bieche et al, 1999).

Our data support the notion that cell lines may be of limited value in the identification of novel markers and drug targets by transcript profiling, because of the limited number of differentially expressed genes. LNCaP cells over-expressed roughly twice as many genes as the hormone-independent, advanced cancer cell line models PC3 and DU-145. Nevertheless, the proportion of gene transcripts undetected in normal tissue, but present in cancer cells, is higher for the cell lines studied than for diseased tissue. Tissue samples exhibited much wider global gene expression activity, with many more transcripts identified as over-expressed. However, this conclusion has several caveats: firstly our array was biased toward differential expression between disease states and normal tissue, rather than towards cell lines. Second, experimental factors such as variation in probe labelling can account for differences in the number of over-expressed genes detected. Thirdly, many of the genes we have identified are associated with host immune response (see Tables 3 and 4) and may not be cancer-specific. The variable proportion of stromal and epithelial cells present in prostatic tissue samples present less of a problem, because all data presented here are compared to mean values for normal prostate tissue or BPH.

We chose to analyse clinical material from TURP and radical prostatectomy specimens rather than needle biopsies. This has the advantage that more material is available, and can be taken from parts of the prostate with macroscopically visible morphological features, adjacent to tissue for which pathology details is available. We believe this approach will anticipate findings in biopsy samples, but lessens the problems associated with heterogeneity in the diseased gland. Ordinarily, 6 needle biopsies taken at different angles are needed to give a good chance of detecting cancer or PIN (Jarmulowicz, 1999). Extra samples can be taken for study, but these are also subject to sampling error and may increase the risk of morbidity in the patient. Our approach has yielded a small number of potential markers which can be justifiably progressed into assessment on biopsy samples in a more convenient, higher throughput format such as RT-PCR.

To keep the physical size of the filter (and therefore the amount of starting mRNA needed) to a minimum, we wished to keep array size within a reasonable limit of 2000 genes. The physical size of the array (~7 × 11 cm) allowed ease of handling, and accommodated a simple first-strand cDNA-labelling method.

Clone sequences were screened prior to arraying using the Incyte sequence databases wherever possible to ensure that Alu or other repeat sequences were not present. Cot-1 DNA was used in some experiments to address problems of repeat sequence hybridization and cross-hybridization. However, comparison of datasets showed this was not a significant problem (data not shown). Certain I.M.A.G.E. clones used were from the Cancer Genome Anatomy Project cDNA libraries Pr1–10 (details are found at http://www.ncbi.nlm.nih.gov/ncicgap/). Whilst these libraries offer the advantage of being derived from microdissected PIN or cancer lesions, clones are made from amplified second strand cDNA, and clone frequency may not reflect true mRNA abundance to the same extent as the Incyte cDNA libraries, generally made by cloning of first strand cDNA. We biased the clone choice on our array towards genes which are expected to express at higher level in cancer, taking clone frequency in cDNA libraries as an indication of their true abundance, but this potential pitfall was kept in mind.

A period of over one year elapsed between the time of clone choice for microarray assembly, and collation of results for this paper, at which time all sequences were checked in the relevant Incyte database for current gene identity. Some clones had been reassigned to a new gene cluster, based on BLAST homology, during this period. This is a result of updating of sequence data from Incyte and new data of public origin which also is assimilated into the Incyte Lifeseq Gold database. Thus, though most array genes were chosen purely on the basis of library frequency, the current database release does not give the same results for some genes. This, combined with an unknown component due to cross-hybridization between closely related cDNA species, probably accounts for the disparity often seen between array data and transcript imaging results. This disparity cautions against inferred associations between genes on the basis of library frequency alone, as has been reported by other groups (e.g. Walker et al, 1999), and strongly suggests that verification of potential markers with highly specific approach is necessary. This work is in progress using RT-PCR methodology.

Assessing the range of gene expression in normal prostate tissue was hindered by the lack of availability of this tissue due to ethical constraints. Mixtures of mRNA from various individuals (as supplied commercially) were the only available source of normal prostate tissue for this study. However, the data from paired filters for 2 different batches of this material was checked for variation of the genes characterized in this study, with the result that over-expression of >1.5 fold above mean was not seen for any gene of interest.

At present, the most specific molecular marker for PIN is absence of GST P1 expression in tumour cells (Brooks et al, 1998). Because PIN cells and normal or BPH cells are often found in the same sample, it is not feasible to test for absence of expression without associated morphological data from microscopy (e.g. via immunohistochemistry). Our shortlist of candidate PIN-associated genes requires verification from more samples, RT-PCR and in situ hybridization, but may lead to a solution to this problem.

There was no obvious difference between global gene expression profiles of cancer and BPH. It was interesting that most of the genes which were over-expressed in cancer were also over-expressed in BPH (data not shown), perhaps reflecting the cellular proliferation which occurs in both states. However, 6 genes were identified as being potential discriminatory markers between BPH and cancer, as detailed in section 3.4. In particular, 3 genes, *DFKZp586D091, laminin B2* and *REA* were detected at high levels in cancer tissue but not detected in either BPH or normal prostate. Further study of the expression of these genes by RT-PCR and in situ hybridization could lead to a rapid molecular diagnostic capable of discriminating cancer from BPH, which avoids extensive histological analysis.

Defining genes that are differentially expressed in normal, BPH, PIN and cancer could lead to molecular testing of biopsies or TUR chips for malignancy. In addition, some of these genes have the potential to be therapeutic drug targets. Further, markers of disease progression in organ-confined cancer patients are needed to plan appropriate therapy, as many of these patients undergo radical prostatectomy without being cured. RT-PCR-based tests in a rapid format could be used during surgery to provide information on likelihood of metastasis. Also, patients could benefit from early therapy if there were molecular indications that metastases were present. Although microarray analysis is not ideal for diagnostic applications, markers thus identified may be validated by further studies, and a panel of markers capable of distinguishing disease states could result. In addition, candidates for serum or immunohistochemical tests can be identified.

In summary we have identified novel associations in prostate cancer for several well characterized genes or full length cDNAs including *PLRP1, JM27, human UbcM2, dynein light intermediate chain 2* and *human homologue of rat sec61* and also novel associations with high-grade PIN, which include: *breast carcinoma fatty acid synthase* and cDNA *DKFZp434B0335*. These genes may prove advantageous in defining prostatic proliferative disease states and may have important diagnostic and therapeutic potential in prostate cancer.

### REFERENCES

Al-Hashim I, Dickinson DP and Levine MJ (1988) Purification, molecular cloning, and sequencing of salivary cystatin SA-1. *J Biol Chem* **263**: 9381–9387

Alon U, Barkai N, Notterman DA, Gish K, Ybarra S, Mack D and Levine AJ (1999) Broad patterns of gene expression revealed by clustering analysis of tumor and normal colon tissues probed by oligonucleotide arrays. *Proc Natl Acad Sci USA* **96**: 6745–6750

Bertucci F, Van Hulst S, Bernard K, Loriod B, Granjeaud S, Tagett R, Starkey M, Nguyen C, Jordan B and Birnbaum D (1999) Expression scanning of an array of growth control genes in human tumor cell lines. *Oncogene* **18**: 3905–3912

Bieche I, Onody P, Laurendeau I, Olivi M, Vidaud D, Lidereau R and Vidaud M (1999) Real-time reverse transcription-PCR assay for future management of ERBB2-based clinical applications. *Clin Chem* **45** (8 Pt 1): 1148–1156

Brooks JD, Weinstein M, Lin X, Sun Y, Pin SS, Bova GS, Epstein JI, Isaacs WB and Nelson WG (1998) CG island methylation changes near the GSTP1 gene in prostatic intraepithelial neoplasia. *Cancer Epidemiol Biomarkers Prev* **7**: 531–536

Buckbinder L, Talbott R, Seizinger BR and Kley N (1994) Gene regulation by temperature-sensitive p53 mutants: identification of p53 response genes. *Proc Natl Acad Sci USA* **91**: 10640–10644

 © 2001 Cancer Research Campaign

Burckzac JD, Cafferkey R and Wilkinson FE (1998) Leveraging genomics for the discovery of diagnostic markers. *J Clin Ligand Assay* **21**: 47–57
Catalona WJ, Smith DS, Ratcliff TL and Basler JW (1993) Detection of organ-confined prostate cancer is increased by prostate specific antigen-based screening. *JAMA* **270**: 948–954
Church GM and Gilbert W (1984) Genomic sequencing. *Proc Natl Acad Sci USA* **81**: 1991–1995
Crawford DE, DeAntoni E and Ross CA (1996) The role of prostate-specific antigen in the chemoprevention of prostate cancer. *J Cell Biochem* **25S**: 149–155
da Silva AJ, Li Z, de Vera C, Canto E, Findell P and Rudd CE (1997) Cloning of a novel T-cell protein FYB that binds FYN and SH2-domain-containing leukocyte protein 76 and modulates interleukin 2 production. *Proc Natl Acad Sci USA* **94**: 7493–7498
Dalton S and Treisman R (1992) Characterization of SAP-1, a protein recruited by serum response factor to the c-fos serum response element. *Cell* **68**: 597–612
Didsbury JR, Ho YS and Snyderman R (1987) Human Gi protein alpha-subunit: deduction of amino acid structure from a cloned cDNA. *FEBS Lett* **211**: 160–164
Giller T, Buchwald P, Blum-Kaelin D and Hunziker W (1992) Two novel human pancreatic lipase related proteins, hPLRP1 and hPLRP2. Differences in colipase dependence and in lipase activity. *J Biol Chem* **267**: 16509–16516
Gleason D (1993) Histological grading of prostate cancer: a perspective. *Human Pathology* **23**: 273–279
Golub TR, Slonim DK, Tamayo P, Huard C, Gaasenbeek M, Mesirov JP, Coller H, Loh ML, Downing JR, Caligiuri MA, Bloomfield CD and Lander ES (1999) Molecular classification of cancer: class discovery and class prediction by gene expression monitoring. *Science* **286**: 531–537
Gong W, Emanuel BS, Collins J, Kim DH, Wang Z, Chen F, Zhang G, Roe B and Budarf ML (1996) A transcription map of the DiGeorge and velo-cardio-facial syndrome minimal critical region on 22q11. *Hum Mol Genet* **5**: 789–800
Haggman MJ, Macoska JA, Wojno KJ and Oeserling JE (1997) The relationship between prostatic intraepithelial neoplasia and prostate cancer: critical issues. *J Urol* **158**: 12–22
Hegarty NJ, Fitzpatrick JM, Richie JP, Scardino PT, deVere White RW, Schroder FH and Coffey DS (1999) Future prospects in prostate cancer. *The Prostate* **40**: 261–268
Imataka H, Olsen HS and Sonenberg N (1997) A new translational regulator with homology to eukaryotic translation initiation factor 4G. *EMBO J* **16**: 817–825
Jarmulowicz MR (1999) The role of pathology in biopsy, diagnosis and management of prostate cancer. In: *Textbook of Prostate Cancer*, Kaisary AV, Murphy GP, Denis L and Griffiths K (eds) pp 17–33. Martin Dunitz: London
Jaiswal AK, Gonzalez FJ and Nebert DW (1985) Human dioxin-inducible cytochrome P1-450: complementary DNA and amino acid sequence. *Science* **228**: 80–83
Jenny RJ, Pittman DD, Toole JJ, Kriz RW, Aldape RA, Hewick RM, Kaufman RJ and Mann KG (1987) Complete cDNA and derived amino acid sequence of human factor V. *Proc Natl Acad Sci USA* **84**: 4846–4850
Jones WG and Smith PH (1994) Cancer of the Prostate. In: *Manual of Clinical Oncology*, sixth edition, Love et al (eds) pp 409–418. Springer-Verlag: Berlin
Kahn S, Yamamoto F, Almoguera C, Winter E, Forrester K, Jordano J and Perucho M (1987) The c-K-ras gene and human cancer. *Anticancer Res* **7(4A)**: 639–652
Kallunki T, Ikonen J, Chow LT, Kallunki P and Tryggvason K (1991) Structure of the human laminin B2 chain gene reveals extensive divergence from the laminin B1 chain gene. *J Biol Chem* **266**: 221–228
Kamine J, Elangovan B, Subramanian T, Coleman D and Chinnadurai G (1996) Identification of a cellular protein that specifically interacts with the essential cysteine region of the HIV-1 Tat transactivator. *Virology* **216**: 357–366
Kay R, Rosten PM and Humphries RK (1991) CD24, a signal transducer modulating B cell activation responses, is a very short peptide with a glycosyl phosphatidylinositol membrane anchor. *J Immunol* **147**: 1412–1416
Landis SH, Murray T, Bolden S and Wingo PA (1998) Cancer statistics. *Cancer J Clin* **48**: 6–29
Menon M (1997) Predicting biological aggressiveness in prostate cancer-desperately seeking a marker. *J Urol* **157**: 228–229
Montano MM, Ekena K, Delage-Mourroux R, Chang W, Martini P and Katzenellenbogen BS (1999) An estrogen receptor-selective coregulator that potentiates the effectiveness of antiestrogens and represses the activity of estrogens. *Proc Natl Acad Sci USA* **96**: 6947–6952
Nomiyama H, Fukuda S, IioM, Tanase S, Miura R and YoshieO (1999) Organization of the chemokine gene cluster on human chromosome 17q11.2 containing the genes for CC chemokine MPIF-1, HCC-2, HCC-1, LEC, and RANTES. *J Interferon Cytokine Res* **19**: 227–234
Reeves WH and Sthoeger ZM (1989) Molecular cloning of cDNA encoding the p70 (Ku) lupus autoantigen. *J Biol Chem* **264**: 5047–5052
Ren Y, Busch RK, Perlaky L and Busch H (1998) The 58-kDa microspherule protein (MSP58), a nucleolar protein, interacts with nucleolar protein p120. *Eur J Biochem* **253**: 734–742
Rokeach LA, Haselby JA, Meilof JF, Smeenk RJ, Unnasch TR, Greene BM and Hoch SO (1991) Characterization of the autoantigen calreticulin. *J Immunol* **147**: 3031–3039
Sagar R (1997) Expression genetics in cancer: shifting the focus from DNA to RNA. *Proc Natl Acad Sci USA* **94**: 952–955
Screaton GR, Caceres JF, Mayeda A, Bell MV, Plebanski M, Jackson DG, Bell JI and Krainer AR (1995) Identification and characterization of three members of the human SRRT family of pre-mRNA splicing factors. *EMBO J* **14**: 4336–4349
Scott IC, Halila R, Jenkins JM, Mehan S, Apostolou S, Winqvist R, Callen DF, Prockop DJ, Peltonen L and Kadler KE (1996) Molecular cloning, expression and chromosomal localization of a human gene encoding a 33 kDa putative metallopeptidase (PRSM1). *Gene* **174**: 135–143
Srivastava M, McBride OW, Fleming PJ, Pollard HB and Burns AL (1990) Genomic organization and chromosomal localization of the human nucleolin gene. *J Biol Chem* **265**: 14922–14931
Talalay P (1989) Mechanisms of induction of enzymes that protect against chemical carcinogenesis. *Adv Enzyme Regul* **28**: 237–250
Walker MG, Volkmuth W, Sprinzak E, Hodgson D and Klinger T (1999) Prediction of gene function by genome-scale expression analysis: prostate cancer-associated genes. *Genome Res* **9**: 1198–1203
Wang K, Gan L, Jeffery E, Gayle M, Gown AM, Skelly M, Nelson PS, Ng WV, Schummer M, Hood L and Mulligan J (1999) Monitoring gene expression profile changes in ovarian carcinomas using cDNA microarray. *Gene* **229**: 101–108
Zazzi H, Nikoshkov A, Hall K and Luthman H (1998) Structure and transcription regulation of the human insulin-like growth factor binding protein 4 gene (IGFBP4) *Genomics* **49**: 401–410
Zhang L, Zhoe W, Velculescu VE, Kern SE, Hruban RH, Hamilton SR, Vogelstein B and Kinzler KW (1997) Gene expression profiles in normal and cancer cells. *Science* **276**: 1268–1272

© 2001 Cancer Research Campaign
